# Identifying cancer tissue-of-origin by a novel machine learning method based on expression quantitative trait loci

**DOI:** 10.3389/fonc.2022.946552

**Published:** 2022-08-09

**Authors:** Yongchang Miao, Xueliang Zhang, Sijie Chen, Wenjing Zhou, Dalai Xu, Xiaoli Shi, Jian Li, Jinhui Tu, Xuelian Yuan, Kebo Lv, Geng Tian

**Affiliations:** ^1^ Gastroenterology Center, The Second People’s Hospital of Lianyungang, Lianyungang, China; ^2^ Lianyungang Clinical College of Xuzhou Medical University, Lianyungang, China; ^3^ The Second People’s Hospital of Lianyungang, Affiliated to Kangda College of Nanjing Medical University, Lianyungang, China; ^4^ Fifth Division of Cancer, Jiamusi Cancer Hospital, Jiamusi, China; ^5^ Department of Mathematics, Ocean University of China, Qingdao, China; ^6^ Department of Oncology, Hiser Medical Center of Qingdao, Qingdao, China; ^7^ Gastrointestinal Surgery, The Second People’s Hospital of Lianyungang, Lianyungang, China; ^8^ Department of Science, Geneis Beijing Co., Ltd., Beijing, China; ^9^ Qingdao Geneis Institute of Big Data Mining and Precision Medicine, Qingdao, China

**Keywords:** cancer of unknown primary, tissue-of-origin, expression quantitative trait loci, XGBoost, TCGA, GEO

## Abstract

Cancer of unknown primary (CUP) refers to cancer with primary lesion unidentifiable by regular pathological and clinical diagnostic methods. This kind of cancer is extremely difficult to treat, and patients with CUP usually have a very short survival time. Recent studies have suggested that cancer treatment targeting primary lesion will significantly improve the survival of CUP patients. Thus, it is critical to develop accurate yet fast methods to infer the tissue-of-origin (TOO) of CUP. In the past years, there are a few computational methods to infer TOO based on single omics data like gene expression, methylation, somatic mutation, and so on. However, the metastasis of tumor involves the interaction of multiple levels of biological molecules. In this study, we developed a novel computational method to predict TOO of CUP patients by explicitly integrating expression quantitative trait loci (eQTL) into an XGBoost classification model. We trained our model with The Cancer Genome Atlas (TCGA) data involving over 7,000 samples across 20 types of solid tumors. In the 10-fold cross-validation, the prediction accuracy of the model with eQTL was over 0.96, better than that without eQTL. In addition, we also tested our model in an independent data downloaded from Gene Expression Omnibus (GEO) consisting of 87 samples across 4 cancer types. The model also achieved an f1-score of 0.7–1 depending on different cancer types. In summary, eQTL was an important information in inferring cancer TOO and the model might be applied in clinical routine test for CUP patients in the future.

## Introduction

About 5% of cancer patients could not be diagnosed with regular clinical and pathological examinations, including medical history inquiry, physical examination, blood routine examination, biochemical examination, urine routine examination, stool routine examination, occult blood test, chest, abdomen and pelvic CT, and immunohistochemical examination (https://www.mskcc.org/cancer-care/types/cancer-unknown-primary-origin). This kind of cancer is called cancer of unknown primary (CUP), which is commonly treated by broad-spectrum chemotherapy with a usually bad prognosis. A landmark study suggested that therapy targeting primary lesion could significantly improve the survival of patients (1). Thus, it is critical to develop novel methods in identifying the tissue-of-origin (TOO) of CUP.

In recent years, many computational methods have been developed for this purpose based on various types of biomarkers (2). For example, He et al. used somatic single-nucleotide polymorphism (SNP) to infer TOO of CUP, which achieved a cross-validation area under curve (AUC) of approximately 0.8 (3). To improve the performance, gene expression profiles were introduced by combining a few machine learning methods like XGBoost and random forest (4, 5). In addition, other markers like miRNA and DNA methylation were also used (6, 7). There are also a few studies integrating multiple types of biomarkers, e.g., SNP and gene expression (8) and gene expression and DNA methylation (7). However, the accuracy especially in independent testing datasets is yet to be improved to meet the clinical criteria. A possible way to improve accuracy is to mine the intrinsic association among various types of biomarkers.

Expression quantitative trait locus (eQTL) is a locus that explains the association between SNPs and gene expression levels (9). eQTL analysis is important in revealing the genetic structure of gene expression (10, 11). For practical purposes, eQTLs were divided into cis-eQTL and trans-eQTL according to the distance from SNP to gene transcription (9). As a common definition, cis-eQTLs are denoted in a predefined window of megabase of a genomic sequence, upstream or downstream of the target gene; trans-eQTLs are denoted as any locus located outside the same window or even on different chromosomes (12). Gong et al. also developed the database PancanQTL following a similar approach, defining cis-eQTL and trans-eQTL of 33 cancer types (13). The database has demonstrated the role of genetic variation in tumor development and progression. Additionally, Gibson et al. introduced some prominent eQTL resources and eQTL publications (14, 15).

Though eQTL has been widely used in cancer research, it has not been applied in CUP analysis. In this study, we integrated eQTL into our machine learning model to infer the primary lesion of CUP. Specifically, we first collected cancer-associated eQTLs based on The Cancer Genome Atlas (TCGA) data portal (https://tcga-data.nci.nih.gov/tcga/) and GTEx analysis (http://www.gtexportal.org/home/). Based on the eQTLs, we trained a CUP model using data from TCGA. We validated the performance of our model by cross-validation and independent testing through our collected data from Gene Expression Omnibus (GEO).

## Materials and methods

### Data preparation

In order to obtain cancer-related eQTL, the calculation can be carried out according to the process mentioned in the *Introduction* section. However, in reality, SNP data are usually inaccessible and not easy to download because they are protected. In the work by Gong and Mei et al., they have calculated the cis-eQTLs and trans-eQTLs in 33 cancer types, and created the database PancanQTL, which is an accessible database (http://bioinfo.life.hust.edu.cn/PancanQTL/) to support searching, browsing, and downloading. We downloaded cis-eQTLs for 20 cancers, which have been studied abundantly and have more complete data samples, from PancanQTL for further study.

The training data were downloaded from TCGA, and the test data were downloaded from GEO. The number and proportion of samples for each cancer in the training data and test data are detailed in **Table 1**.

**Table 1:** Data size and proportion.

Training Data from TCGA				
Cancer Type		Amount	Percent	
Breast invasive carcinoma (BRCA)		1,056	13.68%	
Kidney renal papillary cell carcinoma (KIRC)		526	6.81%	
Uterine corpus endometrial carcinoma (UCEC)		516	6.68%	
Thyroid carcinoma (THCA)		500	6.48%	
Lung adenocarcinoma (LUAD)		486	6.29%	
Head and neck squamous cell carcinoma (HNSC)		480	6.22%	
Colon adenocarcinoma (COAD)		451	5.84%	
Brain lower-grade glioma (LGG)		439	5.69%	
Stomach adenocarcinoma (STAD)		415	5.37%	
Prostate adenocarcinoma (PRAD)		379	4.91%	
Bladder urothelial carcinoma (BLCA)		301	3.90%	
Liver hepatocellular carcinoma (LIHC)		294	3.81%	
Ovarian serous cystadenocarcinoma (OV)		261	3.38%	
Squamous cell carcinoma and endocervical adenocarcinoma (CESC)		258	3.34%	
Kidney renal clear cell carcinoma (KIRP)		222	2.88%	
Acute myeloid leukemia (LAML)		173	2.24%	
Glioblastoma multiforme (GBM)		153	1.98%	
Rectum adenocarcinoma (READ)		153	1.98%	
Pancreatic adenocarcinoma (PAAD)		142	1.84%	
Skin cutaneous melanoma (SKCM)		80	1.04%	
Unknown cancer		430	5.57%	
**Testing Data from GEO**
**Cancer Type**	**Amount**			**Percent**
PRAD	44			38.60%
BRCA	25			45.61%
LUAD	1			00.88%
OV	17			14.91%

### Generate MAP files and PED files

Due to the fact that the input files for the next step, “quality control with Plink”, need to be in MAP and PED formats, the raw TCGA data must be converted into MAP and PED files. There are 7 columns of data in the PED file, and the names of each column are as follows: Family ID (if there is no Family information, the Family ID can be replaced by the Individual ID itself), Individual ID, Paternal ID (0 = unknown), Maternal ID (0 = unknown), Phenotype (0 = unknown), sex (1 = male; 2 = female; 0 = unknown), and SNP type data. There are 4 columns in the MAP file, and the data names of each column are as follows: chromosome number (number format, 0 = unknown), SNP name (character or number, note that it should correspond to SNP column in PED file every to each), molar position of chromosome (optional, 0 = unknown), and SNP physical coordinate (position of variant on chromosome). The MAP content can be defined using the following website: https://docs.gdc.cancer.gov/Data/File_Formats/MAF_Format/.

### Correction covariable and quality control analysis

In this step, confounders are corrected and normalized. A confounder can be any unknown variable that affects the correlation measure between the independent and dependent variables (genetic and non-genetic bias) (16). Its purpose is to remove the impact of technical differences such as bench effects. In order to solve these problems, we need correction covariables and quality control. Daniel Fischer summarized some common software (17): The following are common processes and software in cancer.

1. The first three genotyping principal components (PCs): Firstly, we can do quality control analysis with Plink (http://zzz.bwh.harvard.edu/plink/) or synbree (18, 19). Then, we can use GCTA (https://cnsgenomics.com/software/gcta/#Overview) to generate the top 3 PCs.
2. The first 15 expression PEER (Probabilistic Estimation of Expression Residuals) factors: In this step, we can use PEER Programs (https://hpc.nih.gov/apps/peer.html) to generate 15 PEER factors.
3. Gender, tumor stage, age, and other factors.

### eQTL analysis using MatrixQTL

**Figure 1:**
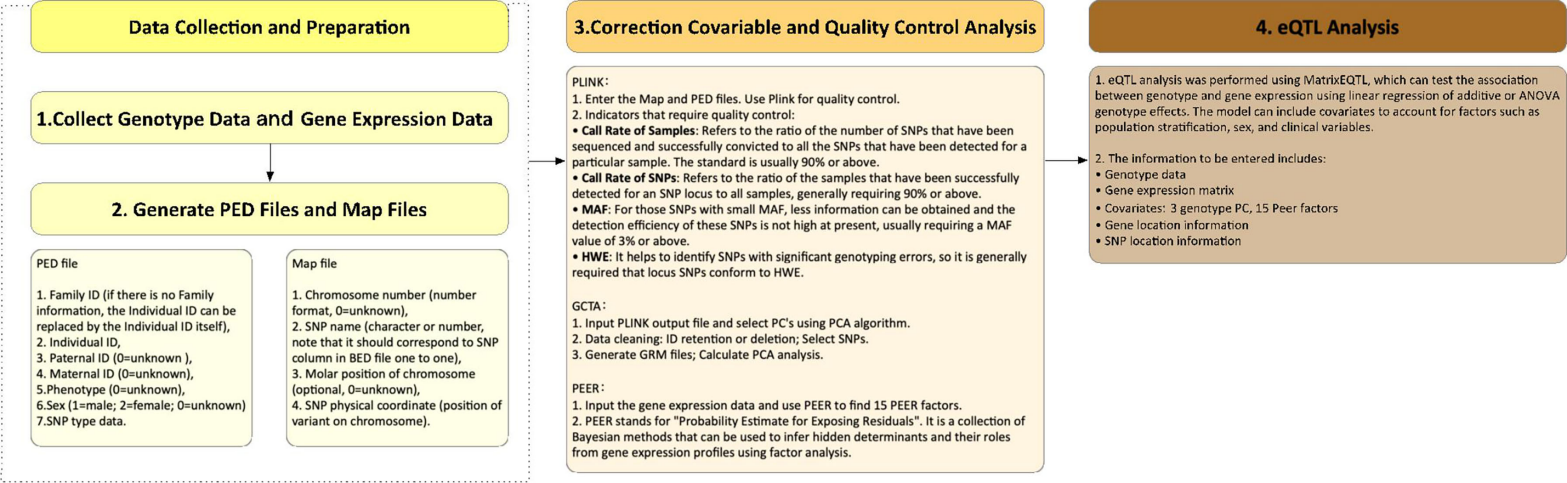
The flow diagram of common eQTL analysis processes. The eQTL data we analyzed were also generated by these processes.

We can also use Merlin, snpMatrix, eMap, FastMap, and other programs (17), but normally, matrixEQTL (http://cran.r-project.org/package=MatrixEQTL) is used for ultra-fast analysis (**Figure 1**). Shabalin et al. developed the program using matrix calculations and explained the statistical principles of the different patterns (**Supplementary Tables 1, 2**).

### Feature selection method

The cis-eQTLs of 20 cancers downloaded from PancanQTL were intersected, and the genes in which these eQTLs were located were identified. Intersect these genes with genes from the training data and test data. The following procedure considered only these genes. Then, random forest was used for feature selection.

Random forest was proposed by Leo Breiman in 2001 (20). It is a kind of integrated learning algorithm that uses a decision tree as a learning machine and uses Bagging (Bootstrap Aggregating) to extract data (21–23). The idea of using random forest to evaluate the importance of features can be summarized as follows: the “contribution” of each feature in each tree in random forest is calculated, and then the “contribution” between features is compared after taking an average value. “Contribution” can often be measured by the Gini Index (formula 4 and formula 5) or OOB (out of bag) (24). The so-called OOB data refer to the data obtained through repeated sampling for training the decision tree whenever the decision tree is established, but about 1/3 of the data are not utilized and do not participate in the establishment of the decision tree (25). This part of data can be used to evaluate the performance of the decision tree and calculate the prediction error rate of the model, which is called OOB data error. This is an unbiased estimate (20).


1 $$Gini\left(D\right)=1-\sum_{i=1}^{\left|C\right|}p_{i}{}^{2}$$

Gini index of *D* is defined under the condition of a known feature *A*:


2 $$Giniindex\left(D,A\right)=\;\sum_{v=1}^{V}\frac{\left|D^{v}\right|}{\left|D\right|}Gini\left(D^{v}\right)$$

Noise interference is randomly added to the features of all samples of OOB data outside the bag (the values of samples at the features can be randomly changed), and the error of data outside the bag is calculated again, which is denoted as *errOOB*2. Assuming there are *N* trees in the forest, the importance of the feature is ∑^​^(*errOOB*2 − *errOOB*1) /*N* .

The reason why this value can explain the importance of the feature is that, if the random noise is added, the accuracy of the OOB data decreases significantly (that is, *errOOB*2 increases), which indicates that this feature has a great influence on the prediction result of the sample, and thus the importance is relatively high.

### Classification method

In this study, we used random forest for feature selection and XGBoost for classifier (5), which was programed by Tian Chen (26). The XGBoost algorithm uses the gradient boosting decision tree algorithm, in which boosting is an ensemble technique where new models are added to correct the errors made by existing models. Models are added sequentially until no further improvements can be made. It uses a gradient descent algorithm to minimize the loss when adding new models. Therefore, gradient boosting makes use of the residual error or error of the previous learner to train the next model and ultimately achieve the predicted effect. The biggest difference between XGBoost and other ensemble learning is that its objective function is added with the regular term after the Taylor expansion, which results in a great increase in its computational speed.

We also used MLP Classfier (multilayer perceptron classifier) for cancer classification. The multilayer perceptron classifier of Kurt Hornik et al. in 1989 was based on the feedforward artificial neural network (ANN) classifier (27). Feedforward neural networks refer to the start of the input layer before receiving only one layer of input and output, and the calculated results to the floor will not give feedback before the whole process can be represented using a directed acyclic graph. The multi-layer perceptron is a full connection between layers, and the layer of any one neuron is connected to the layer of all neurons. In addition to the input and output layers, the MLP Classifier can have multiple hidden layers in the middle. If there is no hidden layer, the problem of linearly separable data can be solved. Here, we use the simplest MLP Classifier (which contains an input layer, a hidden layer, and an output layer structure) to expand the explanation.

***From input layer to hidden layer:*** Since input layer *X*={1, *x*_1_… ,  *x*_*m*_}  to the hidden layer *A*={1,  *a*_1_, … , *a*_*k*_} is fully connected, where element 1 is the bias node, then the output of the hidden layer is  *X*_1_= *f*_1_( *W*_1_ *X*+ *b*_1_), where *W*_1_ is the weight (also known as the connection coefficient); *b*_1_ is offset. The *f* function can be the usual sigmoid or tanh function 3:


3 $$sigmoid\left(x\right)\;=1/\left(1+e^{-x}\right)\;tanh\left(x\right)\;=\left(e^{x}\;-\;e^{-x}\right)\;/\left(e^{x}+e^{-x}\right)\;$$

***From hidden layer to output layer:*** Hidden layer to output layer is a multi-category logistic regression, namely, Softmax regression; thus, the output of the output layer is  *f*_2_( *W*_2_ *X*_1_+ *b*_2_), where *f*_2_ is Softmax function 4.


4 $$Softmax\left(x_{i}\right)\;=\;e^{x_{i}}/\sum_{j=1}^{J}e^{x_{j}}$$

where *x_i_* is the output value of the *i*th node and *J* is the number of output nodes. Obviously, the Softmax function can limit the output value conversion range of multiple classification problems to [0,1], and the sum is 1.

Neural networks have the remarkable ability to make meaning out of complex or imprecise data, and can be used to extract patterns and detect complex trends that neither humans nor other computer technologies can notice. A trained neural network can provide a prediction. Its advantages include the following: MLP is self-adaptive; MLP does not make any comparisons with other probability-based models of functions or other probability-based information considered in its assumptions about potential probability density; and the required decision-making function can be generated directly through training.

## Results

### XGBoost showed better prediction performance than MLP

**Figure 2:**
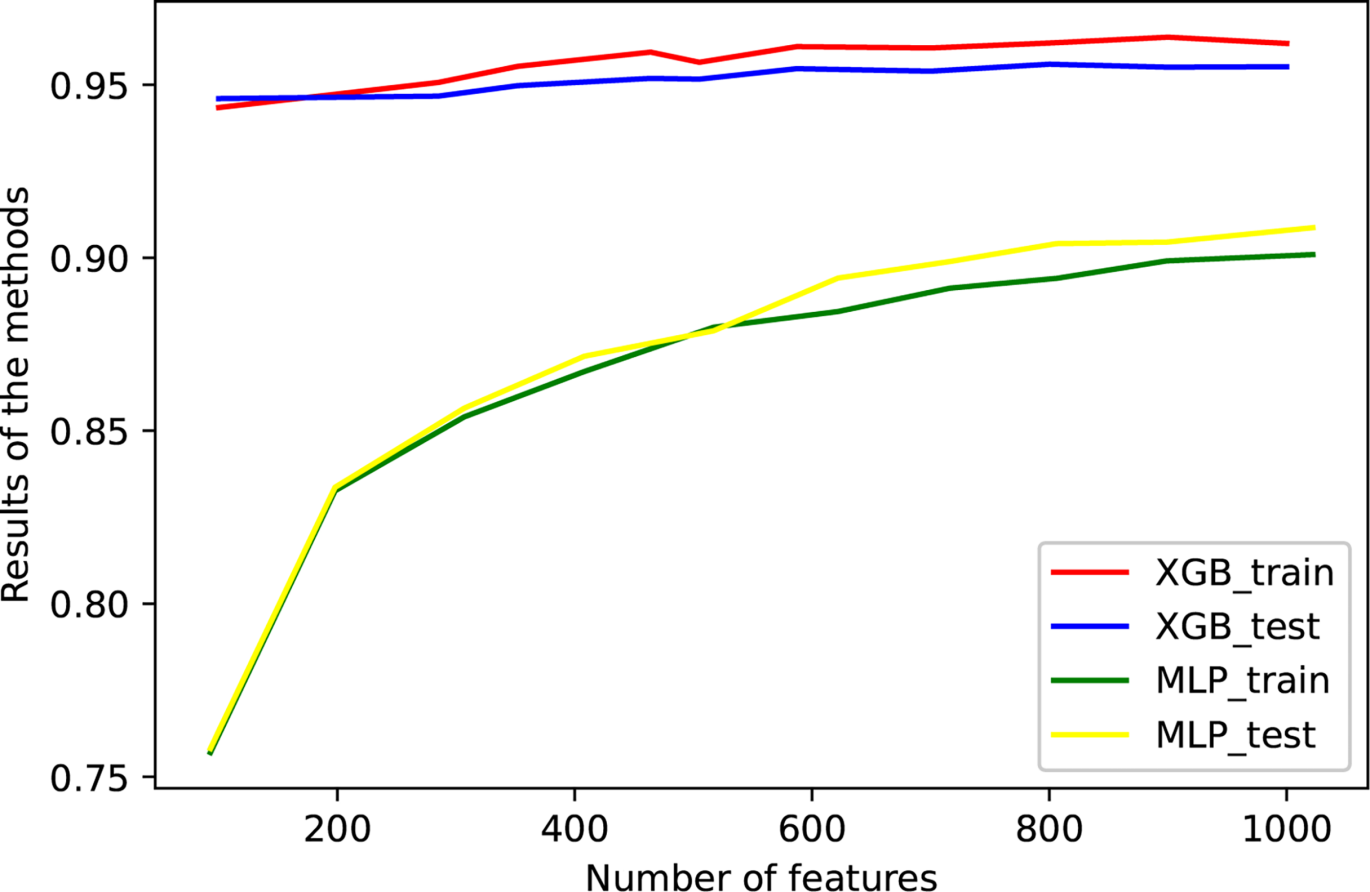
The performance of the model against the number of genes. Tenfold cross-validation was used to train the model, and some data that were not used for training were independently used for testing. XGBoost and MLP were used for classification, respectively. The accuracies of training and verification are shown in this figure.

**Figure 3:**
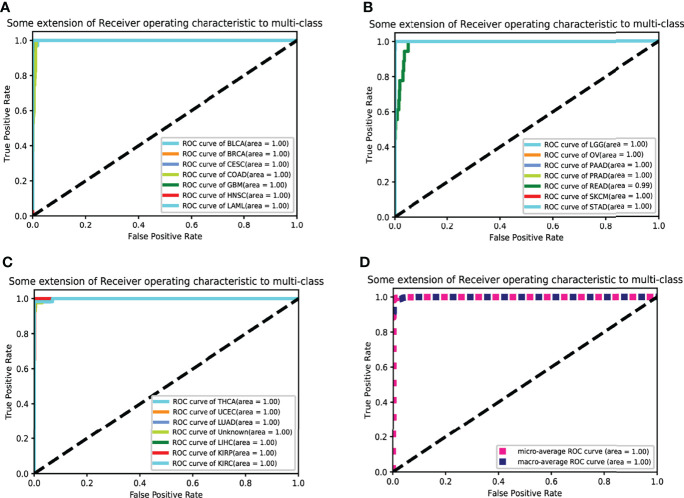
The receiver operating characteristic curve (ROC curve) for classification. Twenty cancer ROC curves of the optimal 10-fold CVs’ results are shown in **(A–C)**. **(D)** The average ROC curve.

The eQTLs of 7,000 samples across 20 types of solid tumors were downloaded from PancanQTL. The genes where these eQTLs were located intersected with the genes in the training data. Following the intersection, the random forest algorithm was used to select the features of these genes, and XGBoost and MLP Classifier were used to classify them. The TCGA data were randomly divided 9:1 and 1/10 was used for testing and 9/10 were used for cross-validation (**Figure 2** and **Table 2**). The results of tenfold cross-validation (10-CV) showed that XGBoost has a higher and more stable accuracy in each feature number. Therefore, XGBoost was used to train TCGA data as a whole (**Figure 3**), and 800 gene features with optimal results in 10-CV were selected to obtain the classifier. Additionally, the trained model was tested independently using 114 samples from four cancer types in a GEO testing data.

**Table 2:** The accuracy of training data and testing data.

Number of features	Accuracy of XGB in training data	Accuracy of XGB in testing data	Accuracy of MLP in training data	Accuracy of MLP in testing data
200	0.943393782383419	0.945990297099496	0.832642487	0.83364232
300	0.950647668393782	0.946705989675118	0.854015544	0.856544482
400	0.956865284974093	0.950883005411232	0.867098446	0.871523024
500	0.956476683937823	0.95160761304501	0.87992228	0.878871727
600	0.9610103626943	0.954631683702029	0.884455959	0.894136587
700	0.960621761658031	0.953910807953061	0.89119171	0.898887898
800	0.962046632124352	**0.955923952480666**	0.894041451	0.904075011
900	**0.963730569948186**	0.955063338378288	0.899093264	0.904507288
1,000	0.961917098445595	0.955207430597308	**0.900906736**	**0.908686169**

Bold values indicate the highest accuracy in each data set.

**Figure 4:**
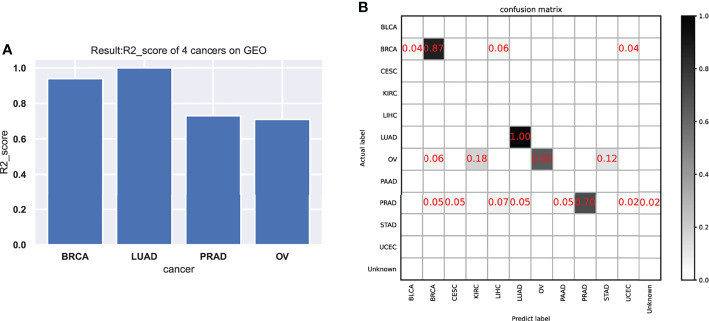
The performance of the model in the testing data. **(A)** The model test results (R2-score) on four cancers. **(B)** The confusion matrix on testing data.

As shown in the results of the test data (**Figure 4** and **Table 3**), the classifier had a better specific recognition capability for BRCA, and the scores of both recall and f1-score were above 90%. We need to improve the recognition of OV and PRAD. The cancer can be isolated alone, or further information can be added based on existing biological pathways.

**Table 3:** The model test results (precision, recall, and f1-score) of 4 cancers on the GEO dataset.

Abbreviation	Precision	Recall	f1-score	Support
PRAD	1	0.729545455	0.83772727	44
BRCA	1	0.940576923	0.97230769	52
OV	1	0.71	0.83	17
LUAD	1	1	1	1
avg/total	1	0.825263158	0.89938596	114

### Top 15 genes in feature selection with each eQTLs

**Figure 5:**
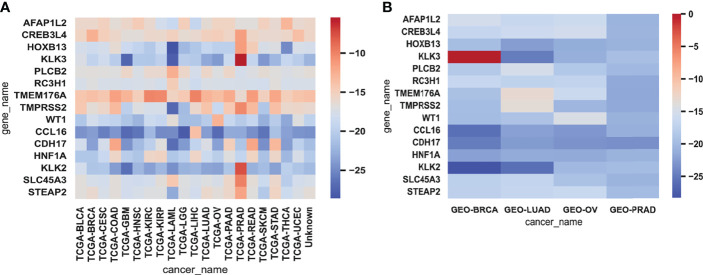
The heatmaps of gene expression. Heatmaps representing the expressions of 15 genes for each cancer sample in the training data **(A)** and testing data **(B)** were averaged and then log-transformed. Red represented high expression and blue represented low expression.

We analyzed 15 genes selected from testing data and training data to reverse-explore the biological implications of their effects on cancer (**Figure 5**). For the *AFFAP1L2* gene, its transcript level in BRCA, KIRP, and LUAD is higher than other cancer types (28). For *CREB3L4*, which is expressed in BRCA and HNSC, the cancer associated with it is prostate cancer (29). *HNF1A* is mainly expressed in BRCA and BCA, leading to familial hepatic adenomas (30). We picked rs1169300 for its maximum magnitude in the presence of this gene; a large study pooling data from 3 Finnish studies totaling over 18,000 individuals concluded that while this SNP is not likely to be causative (relative to cancer), it and one other CRP SNP (rs2464196) are associated with increased risk for lung cancer (31). *KLK3* is expressed in BLCA, BRCA, LIHC, and LUAD, and the gene is highly expressed in cancers such as prostate cancer and breast cancer (32). We picked rs2735839 for its maximum magnitude in the presence of this gene. A study of ~1,800 Caucasian prostate cancer patients concludes that the rs2735839(A) allele is associated with aggressive prostate cancer in general, and more specifically, in Gleason score 7 patients, it is more often associated with being GS 4 + 3 rather than GS 3 + 4 (odds ratio 1.85, CI: 1.31–2.61) (33). *PLCB2* is expressed in BLCA, BRCA, COAD, ESCA, HNSC, KIRC, LGG, and LIHC, and the cancer associated with this gene expression is PRAD (34). *RC3H1* is expressed in BLCA, BRCA, and COAD, and diseases associated with RC3H1 include immune dysregulation and systemic hyperinflammation syndrome and angioimmunoblastic T-cell lymphoma (35). The *TMEM176A* gene is present in BLCA and is highly expressed in liver cancer (36). *TMPRSS2* is expressed in BRCA, GBM, LGG, LIHC, and other cancer types; the *p*-value is the highest in LGG (37). *WT1* is expressed in BRCA, LUAD, and HNSC, with the highest t-stat in BRCA (38). *CCL16* is expressed in STAD, PRAD, and LIHC, which is more obvious in breast cancer. *CDH17* is expressed in BRCA, HNSC, and a gene in metanephric adenoma and gastric cancer (39, 40). *HOXB13* maintains a relatively high transcript level in the adult prostate. We picked rs138213197, which is an SNP in the homeobox transcription factor *HOXB13* gene located in a cluster of HOX genes on ch 17q21–22 (41). Overall, rs138213197(T) was reported to lead to a 20-fold higher risk for prostate cancer, based on having been observed in 72 of ~5,000 patients but in only 1 person out of 1,400 controls (thus, overall odds ratio 20.1, CI: 3.5–803.3, *p* = 8.5 × 10^−7^) (30). *KLK2* is mainly expressed in PRAD and KIRC, resulting in prostate cancer. *SLC45A3* is mainly expressed in BRCA and KIRC, resulting in prostate cancer (42). *STEAP2*, similar to *SLC45A3*, also causes prostate cancer (42).

### Enrichment analysis

**Figure 6:**
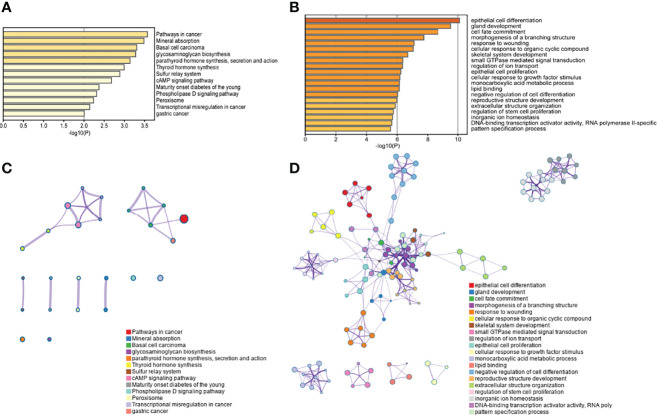
The enrichment analysis display. **(A)** KEGG enrichment histogram. The pathways of 800 genes’ enrichment were demonstrated (*p*< 0.01). **(B)** GO enrichment histogram. The top 20 pathways with 800 genes were demonstrated (*p*< 0.01). The pathway association networks of KEGG and GO are shown in **(C)** and **(D)**. In the networks, each node represented a pathway, and the edges between nodes represented the existence of common genes between pathways.

The top 800 genes that made the testing data the most accurate were selected for enrichment analysis with the Gene Ontology (GO) database and the Kyoto Encyclopedia of Gene and Genomes (KEGG) database by Metascape. The results indicated that these genes were significantly enriched in pathways in cancer, especially in gastric cancer and basal cell carcinoma (**Figures 6A, C**). The KEGG pathway of basal cell carcinoma contained KEGG functional sets of Hedgehog (Hh) signaling, where abnormalities in the Hh signaling pathway have been reported to be associated with divergent cancers (43). The pathway of glycosaminoglycan biosynthesis (GAG) is also significantly enriched in this study. GAG plays multiple regulatory roles in tumor-related angiogenesis, coagulation, invasion, and metastasis (6, 44). Sulfur metabolism and peroxisome are also significantly enriched, both of which are related to the metabolic disorders of cancer (45, 46).

The results of GO enrichment analysis (**Figures 6B, D**) showed that there was significant enrichment of cell adhesion proteins/adhesion involved in cell communication, and the loss of intercellular adhesion may lead to cell escape from the primary lesion and metastasis. Among those, the high expression of plakophilin 2 (PKP2) has been reported to be associated with several human cancers. PKP2 promotes cell proliferation, migration, and invasion by activating the EGFR signaling pathway in LUAD cells (47). Lymphocyte-specific protein tyrosine kinase (LCK) is a key T-cell kinase that is involved in hematologic malignancies (48). In the GO analysis results, there was also significant enrichment of “proto-oncogene vav”, which is a human oncogene derived from a locus commonly expressed in hematopoietic cells (48). In addition, tumor necrosis factor (TNF) was also enriched. TNF induces cell survival, apoptosis, and necrosis, and is widely expressed in cancer (49).

## Discussion

CUP is a malignant cancer with a high mortality rate. The study of CUP from the perspective of gene expression and SNP is conducive to the fundamental understanding of the disease and the improvement of treatment.

In previous studies, eQTL has shown tissue specificity (50). eQTL is also used to study cancer risk, development, and treatment response. We have used a novel approach to incorporate cancer-related eQTLs into our cancer tissue traceability model. We extracted genes with cancer-related eQTLs as part of the feature selection process and used the genes with cancer-related eQTLs for subsequent model training. Following feature selection-based eQTL analysis, the number of genes was reduced from 23,366 to 16,717. This significantly improves the prediction and generalization capabilities of the model.

In this model, eQTL is applied to infer tumor origin for the first time, which achieved better performances than using single markers. However, there are a few limitations of this study. Firstly, previous studies suggested that other biomarkers like pathological images are important in cancer diagnosis and prognosis prediction (51–53). It would be interesting to incorporate these biomarkers together with eQTL to infer TOO of CUP. Secondly, the machine learning algorithm used in this study is quite standard. More complicated models might be able to improve the performance as shown elsewhere (54, 55). Finally, the independent testing dataset used in this study is small, and a dataset containing more types of cancers should be curated in the future.

## Conclusion

In this study, we first described the biological basis of eQTL and the commonly used mathematical models, then we discussed the application of eQTL in diseases and cancer, as well as the general use of eQTL in cancer analysis and other software and websites for additional information. We used eQTL to classify cancer. The results of 10-fold cross-validation of TCGA data with different features led to the selection of XGBoost as the optimal model, and the reason for this selection is explained along with its eQTL. Afterward, we discussed the possibility of using other algorithms in eQTL analysis to solve the problems in traditional analysis, and also discussed the use of eQTL analysis for subjects other than mRNA expression.

## Data availability statement

The original contributions presented in the study are included in the article/supplementary material. Further inquiries can be directed to the corresponding authors.

## Ethics statement

Ethical review and approval was not required for the study on human participants in accordance with the local legislation and institutional requirements. Written informed consent for participation was not required for this study in accordance with the national legislation and the institutional requirements.

## Author contributions

KL and GT conceived the project; YM and XZ implemented the experiments and wrote the manuscript; WZ and DX collected data; DX, XS, SC, JL, JT, and XY analyzed the data and revised the manuscript; all authors approved the final manuscript.

## Conflict of interest

Authors GT and XS were employed by Geneis Beijing Co. Ltd and Qingdao Geneis Institute of Big Data Mining and Precision Medicine. XY was employed by Geneis Beijing Co. Ltd.

The remaining authors declare that the research was conducted in the absence of any commercial or financial relationships that could be construed as a potential conflict of interest.

## Publisher’s note

All claims expressed in this article are solely those of the authors and do not necessarily represent those of their affiliated organizations, or those of the publisher, the editors and the reviewers. Any product that may be evaluated in this article, or claim that may be made by its manufacturer, is not guaranteed or endorsed by the publisher.
