# Supplementary material for: Identifying cancer tissue-of-origin by a novel machine learning method based on expression quantitative trait loci
Source: Front Oncol. 2022 Aug 9;12:946552. doi: 10.3389/fonc.2022.946552 (PMC9396384; doi:10.3389/fonc.2022.946552)
Supplement: Supplementary file 1 [file Table_1.docx]

**Supplementary Table 1.** Reference Websites

| **Software** | **Function** | **Platform** | **URL** |
| --- | --- | --- | --- |
| EMMA | Population structure | R | http://mouse.cs.ucla.edu/emma/ |
| AFFYGG | Allelic confounders | R | github.com/DannyArends/GBIC/tree/master/AffyGG |
| SVA | Population structure；batch effect | R | bioconductor.org/packages/release/bioc/html/sva.html |
| Affy | Normalization | R | bioconductor.org/packages/release/bioc/html/affy.html |
| Merlin | eQTL analysis | Command line | csg-old.sph.umich.edu//abecasis/Merlin/ |
| Pseudomarker | eQTL analysis | Matlab | churchill.jax.org/software/archive/ pseudomarker.shtml |
| snpMatrix | eQTL analysis | R | bioconductor.org/packages/2.4/bioc/ html/snpMatrix.html |
| eMap | eQTL analysis | R | bios.unc.edu/~weisun/software.htm |
| J/qtl | eQTL analysis | GUI | churchill.jax.org/software/jqtl.shtml |
| MapQTL | eQTL analysis | MS-Windows | https://kyazma.nl/index.php/MapQTL/ |
| GridQTL | eQTL analysis | Browser | gridqtl.org.uk |
| QTLMap | eQTL analysis | Command line | forge-dga.jouy.inra.fr/projects/qtlmap |
| Matrix eQTL | eQTL analysis | R and others | bios.unc.edu/research/genomic_software/ Matrix_eQTL/ |
| PLINK | eQTL analysis | Command line | www.cog-genomics.org/plink2 |
| FastQTL | eQTL analysis | Command line | http://fastqtl.sourceforge.net/ |
| R/qtl | eQTL analysis | R | rqtl.org/ |

**Supplementary Table 2.** Prominent eQTL resources (Gibson et al., 2015)

| **Resource** | **URL** | **Nature of data** |
| --- | --- | --- |
| GeneVar | http://www.sanger.ac.uk/resources/software/genevar/ | eQTL visualization tools |
| Geuvadis | http://www.ebi.ac.uk/Tools/geuvadis-das/ | HapMap LCL eQTLs |
| Blood eQTL | http://genenetwork.nl/bloodeqtlbrowser/ | Blood eQTL meta-analysis |
| GTEx Portal | http://www.gtexportal.org/home/ | Multi-tissue eQTL study |
| NCBI | http://www.ncbi.nlm.nih.gov/projects/gap/eqtl/index.cgi | Searchable database of GTEx |
| Chicago eQTL | http://eqtl.uchicago.edu/Home.html | eQTLs with genomic features |
| Pickrell laboratory | http://gwas-browser.nygenome.org/ | eQTLs with GWAS association |
